# Self-Generated or Cue-Induced—Different Kinds of Expectations to Be Considered

**DOI:** 10.3389/fpsyg.2017.00053

**Published:** 2017-01-24

**Authors:** Maike Kemper, Robert Gaschler

**Affiliations:** ^1^Interdisciplinary Research Cluster on Image, Knowledge, Gestaltung, Department of Psychology, Humboldt-Universität zu BerlinBerlin, Germany; ^2^Interdisciplinary Research Cluster on Image, Knowledge, Gestaltung, Department of Psychology, FernUniversität in HagenHagen, Germany

**Keywords:** stimulus expectation, self-generated expectations, predictions, cue-induced expectations, violation of expectation

## Differences between self-generated and cue-induced expectations

Expectations can help humans to adequately prepare for action. Cognitive psychology has inspired studies on the influence of expectations on the course of scientific discovery (Klein and Roodman, 2005; Rzhetsky et al., 2006; Brewer, 2012). Violations of expectations in research often fail to provoke changes in theorizing and research practices. While expectations have been dissociated from other processes such as automatic response activation (Perruchet et al., 2006), relatively little attention has been devoted to reflecting on the different forms of expectation or different methods used to study expectations (and their violations). In this opinion paper, we highlight some early work (Acosta, 1982) and later contributions that have the potential to violate researchers' expectations on what seems the most suitable methodology for operationalizing expectations in the cognitive psychology lab.

In behavioral research, expectations are most often operationalized by assessing performance differences between trials in which expectations are met and trials in which expectations are violated. Neurophysiological data can assess dynamics before stimulus onset (e.g., Mattler et al., 2006; Kemper et al., 2012), and the mismatch effect shows that response times are faster and error rates lower for expected events (compared to when an expectation is violated). This can be demonstrated for expectations about stimuli (Posner and Snyder, 1975; Mattler, 2004), as well as to-be-performed tasks in task switching studies (Rogers and Monsell, 1995; Meiran, 1996). Furthermore, expectations can modulate the impact of cognitive conflicts (Duthoo et al., 2013, for a study on expectations in the Stroop task). The Gratton effect is a change in the strength of a conflict effect depending on the amount of cognitive conflict in previous trials. It has been described as an expectation effect (Gratton et al., 1992; see also Botvinick et al., 1999; Braver, 2012; but see e.g., Mayr et al., 2003; Schmidt and Weissman, 2016, for alternative interpretations).

Many studies use cues to induce stimulus expectations (Posner and Snyder, 1975; Shulman et al., 1999; Mattler, 2004; Oswal et al., 2007) and task expectations (Rogers and Monsell, 1995; Meiran, 1996). Other methods of inducing expectations include presentation of subliminal stimuli (Kunde, 2004) or irrelevant flankers (Nattkemper et al., 2010; Ziessler and Nattkemper, 2011). At first sight inducing expectations seems to offer a greater degree of experimental control compared to allowing participants to form their own expectations. By inducing expectations, experimenters can determine in advance how often which cue is used and how often the upcoming event violates vs. matches the expectation that the cue should induce. However, studies of stimulus expectations show stronger behavioral (Acosta, 1982) as well as EEG effects (Kemper et al., 2012) for self-generated compared to cue-induced expectations. This suggests that self-generated expectations might nonetheless be preferable, as they induce larger, and therefore more easily detectable, effects.

In a self-generated expectation condition, participants are prompted to verbalize their expectation (e.g., “shape?”). They verbalize which of the stimuli from the set (e.g., “circle”) they are expecting to appear in the current trial. In a cuing variant, the participants are shown a picture of a circle or the word “circle” (cf. Kemper et al., 2012) and verbalize it. Next, the stimulus is shown and the response is collected. One early example hinting at a qualitative difference between self-generated vs. cue-induced expectations was reported by Acosta (1982). For mismatches, larger stimulus set sizes led to longer response times, for self-generated and cue-induced expectations alike, whereas for match trials, set size effects differed between cue-induced and self-generated expectations. Reaction times for stimuli that matched the cue were longer if more stimuli were used. For self-generated expectations, set size had only a minor influence. Presumably, the expected stimulus was strongly activated regardless of whether there were many or few alternative stimuli. In addition, Acosta (1982) reported evidence that violations of self-generated expectations have a stable effect when prolonging the interval between generation of the expectation and stimulus presentation whereas cue-induced expectations diminish relatively quickly for prolonged intervals. Stronger effects of violations of self-generated compared to cue-induced expectations have not only been obtained for expectations of stimuli. They were also found when expectations concerned a more abstract level of task processing, such as the conflict level of the upcoming trial (e.g., expecting a congruent vs. an incongruent Stroop trial; Kemper et al., 2016). Specifically, expecting the repetition of a congruent trial led to faster processing, while expecting conflict did not enhance performance (e.g., Duthoo et al., 2013). A modulation was found for self-generated expectations only.

Stronger effects of self-generated compared to cue-induced expectations can be attributed to (a) differences in strength and (b) likelihood of engagement. While there is evidence that cues can be ignored (especially in case of low validity; cf. Alpay et al., 2009), even chance-level validity leads to strong effects of self-generated expectations (e.g., Acosta, 1982; Kemper et al., 2012, 2016; Gaschler et al., 2014). This suggests that self-generated expectations cannot be ignored (see Schwager et al., 2016, for a current test of boundary conditions), whereas participants presumably fail to attend to or use cues of low validity in many of the trials. Based on this, and on the lack of a set size effect reported by Acosta (1982), Gaschler et al. (2014) suggested that the object of expectation becomes represented in the focus of attention in working memory (cf. Oberauer et al., 2013) in the case of self-generated expectations (but only occasionally so in the case of cues). This representation is accessible for verbal report, which implies that verbalizations are a rather direct measure of self-generated expectations. The stimulus representation that is activated more strongly than the others (if only by a small margin) can be selected for report.

More specifically, there is evidence for the assumption that this privileged form of representation is a by-product of self-assessing what one is currently expecting. Strong RT benefits for the stimulus that one says one is *not* expecting (Hacker and Hinrichs, 1979) or is expecting second *most* (Hacker and Hinrichs, 1974) suggest that the focus of attention in working memory is filled with the object one considers when self-assessing what one is currently expecting. Thus, researchers should take into account that self-generated verbalized expectations might not only serve as a measure of expectation, but also as a means of boosting expectation effects. Based on the evidence gathered so far, it is difficult to determine whether a stronger effect is in general desirable to increase the internal validity of the experiment or whether this comes at the cost of results that are only representative for the specific situation in which participants are required to form and verbalize an expectation. Studies that directly compare self-generated expectations when participants are either triggered to form them or can form them spontaneously have so far not been conducted. They might become possible using neurophysiological multivariate pattern recognition to trace expectations (cf. Cichy et al., 2014).

## Restricted influence of expectation violations on self-generated expectations

Self-generated expectations allow experimenters to track how violations of expectations influence the formation of future expectations. For example, expectations about the conflict level of an upcoming trial are highly dependent on the conflict level of the previous run of trials. For instance, participants expect a repetition of a conflict trial after one single conflict trial. However, the more conflict trials that have occurred in a row, the stronger participants show a gambler's fallacy and expect a congruent trial next (Jiménez and Méndez, 2013, 2014; Kemper et al., 2016). It is possible that violations of expectations operate like a conflict cue for processing in the upcoming trial. Exploring this possibility might help to further understand the differences between cue-induced and self-generated expectations.

In addition to effects of violation of expectation on future expectations that depend on the last (few) trial(s), stimulus probability influences the overall percentage in which participants predict each stimulus (e.g., Kemper et al., 2012). The phenomenon of probability matching (e.g., Umbach et al., 2012) suggests that self-generated expectations are not strategically chosen to optimize performance. For instance, if Stimulus A is 70% likely and Stimulus B is only 30% likely, probability matching means that participants will anticipate Stimulus A on 70% of the trials, and anticipate Stimulus B on 30% of the trials, even though to optimize performance (by optimizing the number of trials in which expectation and stimulus match) the best solution is to anticipate Stimulus A on 100% of the trials. In principle, participants could exclusively verbalize that they are expecting the frequent stimulus. This would maximize the number of match trials and should improve performance. Such strategic effects would undermine the credibility of verbalizations as a valid measure of expectations. However, participants match their expectations to the probabilities of stimuli instead of minimizing expectation violations (e.g., Kemper et al., 2012; Umbach et al., 2012). Expectations seem to be influenced by and to reflect stimulus frequencies. Future research should explore whether this influence is in part the result of (other) strategic effects. For example, participants might aim to match verbalization frequencies to stimulus frequencies in an attempt to obtain match trials even for the infrequent stimuli. In addition, probability matching can be an effect of the search for patterns (Gaissmaier and Schooler, 2008).

So far, the evidence for strategic effects is limited. The mismatch effect is stable with practice and of similar strength for frequent and infrequent stimuli, even though violations of expectations are much more likely for infrequent than for frequent stimuli (e.g., Umbach et al., 2012). Frequent violation of an expectation does not influence how strongly that expectation is relied upon in future trials. However, validity might have a different effect on cue induced than on self-generated expectations. Cues show larger mismatch effects when they are relatively valid (i.e., when the expectation is violated less often) and mismatch effects become very small for cues with low validity (e.g., Vossel et al., 2006).

## Conclusions for research on (the violation of) expectations

Self-generated expectations show stronger effects than cue-induced expectations in a number of experimental setups, and measure expectations more effectively relative to cues. People still rely on self-generated expectations even if they are violated often (e.g., are of low validity in a long experiment). We suggest that researchers should take into account that the choice between self-generated and cue-induced expectations entails a tradeoff between the strength of the expectation effect and the degree of experimental control over expectations in individual trials. In addition, since internally-generated expectations may differ qualitatively from those induced by cues, it cannot be taken for granted that results obtained with one method can be generalized to situations involving the other.

## Author contributions

MK and RG have written this article together. They have both contributed substantial, direct and intellectual contribution to the work, and approved it for publication.

## Funding

This work was supported by the Interdisciplinary Research Cluster Image, Knowledge, Gestaltung (EXC1027/1), at Humboldt-Universität zu Berlin, Berlin.

### Conflict of interest statement

The authors declare that the research was conducted in the absence of any commercial or financial relationships that could be construed as a potential conflict of interest.
